# Identification of novel hub genes for Alzheimer’s disease associated with the hippocampus using WGCNA and differential gene analysis

**DOI:** 10.3389/fnins.2024.1359631

**Published:** 2024-03-07

**Authors:** Yang Chen, Zhaoxiang Li, Xin Ge, Huandi Lv, Zuojun Geng

**Affiliations:** ^1^Graduate School, Hebei Medical University, Shijiazhuang, China; ^2^Department of Immunology and Pathogenic Biology, Yanbian University Medical College, Yanji, China; ^3^Science and Education Section, Baoding First Central Hospital, Baoding, China; ^4^Department of Medical Imaging, The Second Hospital of Hebei Medical University, Shijiazhuang, China

**Keywords:** Alzheimer’s disease, comprehensive analysis, WGCNA, hippocampi, biomarker differentially expressed gene (DEG) selection

## Abstract

**Background:**

Alzheimer’s disease (AD) is a common, refractory, progressive neurodegenerative disorder in which cognitive and memory deficits are highly correlated with abnormalities in hippocampal brain regions. There is still a lack of hippocampus-related markers for AD diagnosis and prevention.

**Methods:**

Differently expressed genes were identified in the gene expression profile GSE293789 in the hippocampal brain region. Enrichment analyses GO, KEGG, and GSEA were used to identify biological pathways involved in the DEGs and AD-related group. WGCNA was used to identify the gene modules that are highly associated with AD in the samples. The intersecting genes of the genes in DEGs and modules were extracted and the top ten ranked hub genes were identified. Finally GES48350 was used as a validation cohort to predict the diagnostic efficacy of hub genes.

**Results:**

From GSE293789, 225 DEGs were identified, which were mainly associated with calcium response, glutamatergic synapses, and calcium-dependent phospholipid-binding response. WGCNA analysis yielded dark green and bright yellow modular genes as the most relevant to AD. From these two modules, 176 genes were extracted, which were taken to be intersected with DEGs, yielding 51 intersecting genes. Then 10 hub genes were identified in them: HSPA1B, HSPB1, HSPA1A, DNAJB1, HSPB8, ANXA2, ANXA1, SOX9, YAP1, and AHNAK. Validation of these genes was found to have excellent diagnostic performance.

**Conclusion:**

Ten AD-related hub genes in the hippocampus were identified, contributing to further understanding of AD development in the hippocampus and development of targets for therapeutic prevention.

## 1 Introduction

Alzheimer’s disease (AD) is a complex multi-factorial neurodegenerative disorder with a hidden onset that affects more than 50 million people worldwide (Liu et al., 2022). AD is the leading cause of mortality in dementia and due to its non-curable reality based on current medical development status, it has brought extreme economic burden to patients’ households as well as continuous distress. The main clinical signs and symptoms of AD include subjective cognitive decline (SCD), behavioral change, and dementia (Jessen et al., 2014). However, before clinical symptoms are observed, alterations in neurons, microglia, and astroglia have already driven insidious progression of the disease (De Strooper and Karran, 2016). The preclinical phase was termed as the cellular phase of AD (Scheltens et al., 2021). However, in most cases when AD could be definitively diagnosed, the patient is already in the dementia stage. Recent studies have found that intervention and prevention before the onset of AD may delay or even prevent the occurrence and development of the disease (Feng et al., 2021; McDade et al., 2021). And, current medications and FDA-approved treatments cannot cure AD and AD-related dementia (Fang et al., 2022). Therefore, it is significant to find effective biomarkers for early diagnosis and early prevention of AD.

Researches have confirmed that the precipitating features of AD involve multiple factors, among which most studies believe that AD, as a progressive and extensive neurodegenerative disease, is characterized by extensive gliosis, and the accumulation of amyloid β (Aβ) in the form of extracellular plaques and intracellular neurofibrillary tangles, ultimately leading to neurodegeneration and dementia (Joe and Ringman, 2019). Meanwhile, existing studies have found that excitatory neurotransmitter deficiencies or dysfunctions, as well as cerebrovascular dysfunction, are also involved in the development of AD (Fang et al., 2022). And adult hippocampal neurogenesis (AHN), an important neuroplasticity process, is also involved in the development of AD. A decrease in AHN may lead to hippocampal degeneration manifested by progressive memory loss and even the development of cognitive impairment (Moreno-Jimenez et al., 2021; Kim et al., 2022). In addition to the decrease in AHN, the presence of plaques and tangles in the hippocampus is also strongly associated with cognitive decline. However, there is no consensus on the core cause of cellular dysfunction in AD. Research over the last 5 years has focused on the use of proteomics, genomics and transcriptomics to investigate the pathogenesis of key metabolic pathways and regulators of Alzheimer’s disease (Nativio et al., 2020; Horgusluoglu et al., 2022). In this study we aimed to identify novel hub genes involved in AD development in the hippocampal brain region by differential gene screening combined with WGCNA analysis, providing new targets for clinical diagnosis and treatment of AD.

## 2 Materials and methods

### 2.1 Data extraction and differential gene expression analysis

Gene Expression Omnibus (GEO) is a public functional genomics data repository of high-throughput gene expression data, chips, and microarrays. Two datasets GES29378 and GES48350 related to AD were extracted from the GEO database. We extracted the expression profile data of the hippocampal region tissues in the GES29378 (divided into AD and normal groups). Specific data information is shown in Table 1. The obtained expression profile data of patients have been normalized for total expression and then log_2_FC transformed. Differently expression gens (DEGs) were obtained by using “limma R” packages in R software with |log_2_ Fold Change (FC)| > 0.5 and *p* < 0.05. All samples were corrected by using the “limma” package in the R software (version 4.1.3) (Ritchie et al., 2015). R software was used for statistical analysis, and the difference was statistically significant when *p* < 0.05. Visualization was performed by the “ggplot2 R” package. Volcano plot, heatmap, and PCA plot of the DEGs were generated by using “ggplot2” and “pheatmaps” packages in R software (version 4.1.32).

**Table 1:** Specific information on the source of the data.

Source database	Data type	Data grouping information	Data grouping information
GEO	Transcriptome information	GSE29378	Hippocampal tissue samples from 31 AD patients, 32 normal hippocampal tissue samples
GEO	Transcriptome information	GSE48350	Hippocampal tissue samples from 19 AD patients, 43 normal hippocampal tissue samples

### 2.2 GSEA analysis of samples and functional enrichment analysis of DEGs

The functional enrichment of DEGs was divided into three categories of gene ontology (GO) domain: biological process (BP), cellular component (CC) and molecular function (MF). The KEGG database contains pathway datasets involving biological functions, diseases, chemicals and drugs. We subjected the DEGs of GES29378, modular genes in WGCNA, and intersecting genes to GO and KEGG analysis. In this investigation, these DEGs were analyzed by R langue (cluster profile package [version 3.14.3], Org.hs.eg. DB package [version 3.10.0] (for ID conversion)) (Yu et al., 2012). The detailed data was used ggplot2 package to demonstrate.

GSEA was performed using cluster profile in R (Martinez-Zamudio et al., 2020). This method specified whether the pathways were randomly distributed at the top or bottom of the detected genes. The coefficients of Spearman correlation between genes and sample label were defined as the weight of genes. Statistical significance was assessed by comparing the enrichment score to enrichment results generated from 1,000 random permutations of the gene sets to obtain values (nominal value). GSEA analysis was performed on the expression profiles among samples of GES29378 to identify significant pathways involved in the development of AD.

### 2.3 WGCNA network construction for AD expression profiling

Overall, we calculated the Median Absolute Deviation (MAD) of each gene using the gene expression profiles of GES29378, eliminated the top 50% of genes with the smallest MAD, removed the outlier genes and samples by using the goodSamplesGenes method of the R software package WGCNA, and further constructed a scale-free co-expression network using WGCNA (Langfelder and Horvath, 2008). First, the Pearson correlation matrix and the average linkage method were computed for all paired genes, and then a weighted neighbor-joining matrix was constructed using the function. β is a soft-threshold parameter that emphasizes strong correlations between genes and penalizes weak correlations. After selecting powers of 16, the neighbor-joining matrix was transformed into a topological overlap matrix (TOM), which measures the network connectivity of a gene, defined as the sum of the neighbor-joining matrices of that gene and all other genes assigned to genes of the network, and the corresponding dissimilarity (1-TOM) was calculated. In order to group genes with similar expression profiles into gene modules, average connectivity hierarchical clustering was performed according to the TOM-based similarity metric, with the minimum size (genome) of the gene dendrogram being 30 and the sensitivity set to 3. To further analyze the modules, we computed the similarity of the genes characterizing the modules, selected the cut lines of the module dendrograms, and merged a number of modules. In addition, we merged modules with distances less than 0.25, resulting in 12 co-expression modules, where gray modules were considered to be the set of genes that could not be assigned to any module.

### 2.4 Analysis of trait relationships between modules and AD

Correlations between co-expression modules and clinical traits were estimated based on whether the patient was under AD status, patients’ gender, and different hippocampal regions. Significant co-expression modules highly correlated with traits were identified. Module-trait relationships were calculated using the Pearson correlation test, and significant correlations were considered at *p* < 0.05.

### 2.5 Extraction and characterization of modular genes associated with AD pathological states

Gene screening requires the identification of module membership (MM) to determine the correlation between gene and given module. Therefore, MM values for each gene need to be calculated to identify important genes in the module. Correlation analysis was performed between the gene significance for AD occurrence and the MM of each module gene to test whether the MM is strongly associated with the AD occurrence status. After identifying several modules with the highest correlation (bright yellow module and dark green module), the specific genes in them were extracted. GO and KEGG analyses were also performed in the modules to further analyze the biological signaling pathways involved in the development of AD.

### 2.6 Identification of the hub genes in AD

We extracted intersecting genes from DEGs in AD and modular genes extracted from WGCNA. The extracted intersecting genes were then uploaded into the STRING database to further understand the interactions between the corresponding differential genes and construct protein interactions networks, using the composite score > 0.4 as the cutoff point. The obtained TSV files were downloaded and submitted to Cytoscape software to filter the top ten ranked hub genes by examining the topology of the protein–protein interaction network using the java module cytoHubba in Cytoscape.

### 2.7 External validation of the predictive efficacy of hub genes of AD

To further validate whether the obtained hub genes are diagnostic for the development of AD. We again extracted the AD-related dataset GSE48350, compared the transcript information of RNAseq data in normal hippocampus and hippocampus of AD patients. Then we analyzed the diagnostic ability of the hub gene, plotted the ROC curve using the R package “pROC,” and calculated the area under the ROC curve (AUC). The area under the ROC curve (AUC) was calculated. When the AUC was greater than 0.6, the relative molecule was regarded as a diagnostic marker with a certain degree of accuracy.

## 3 Results

### 3.1 Identification of AD-associated DEGs

**Figure 1:**
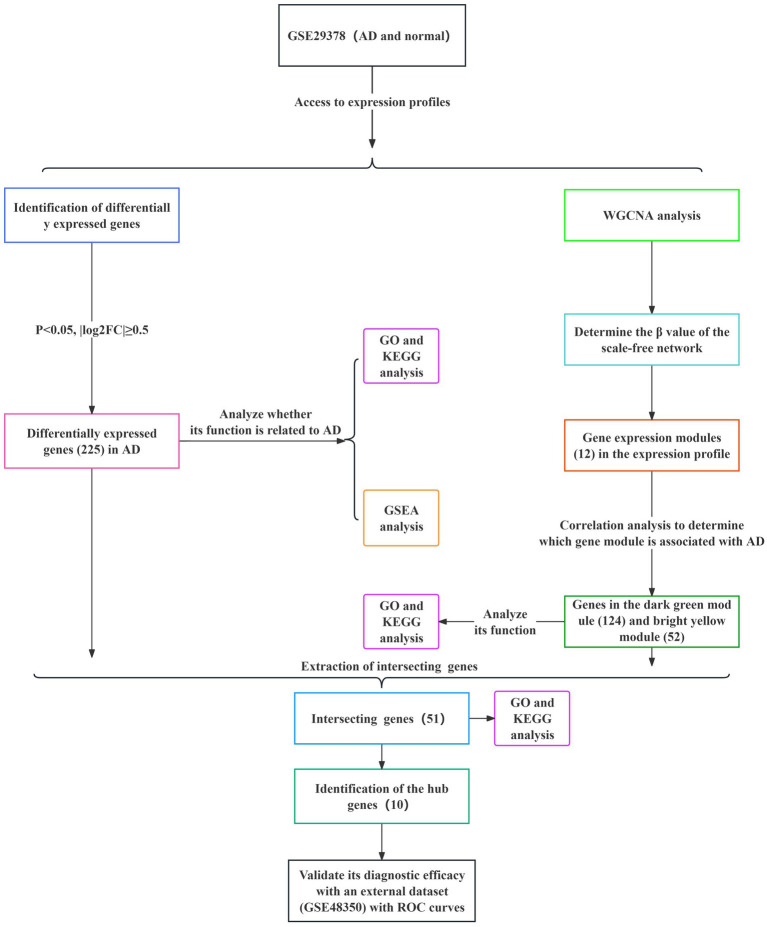
Specific process of analysis. The datasets used for each analysis, as well as the methods used for the analysis, are indicated by different color blocks.

**Figure 2:**
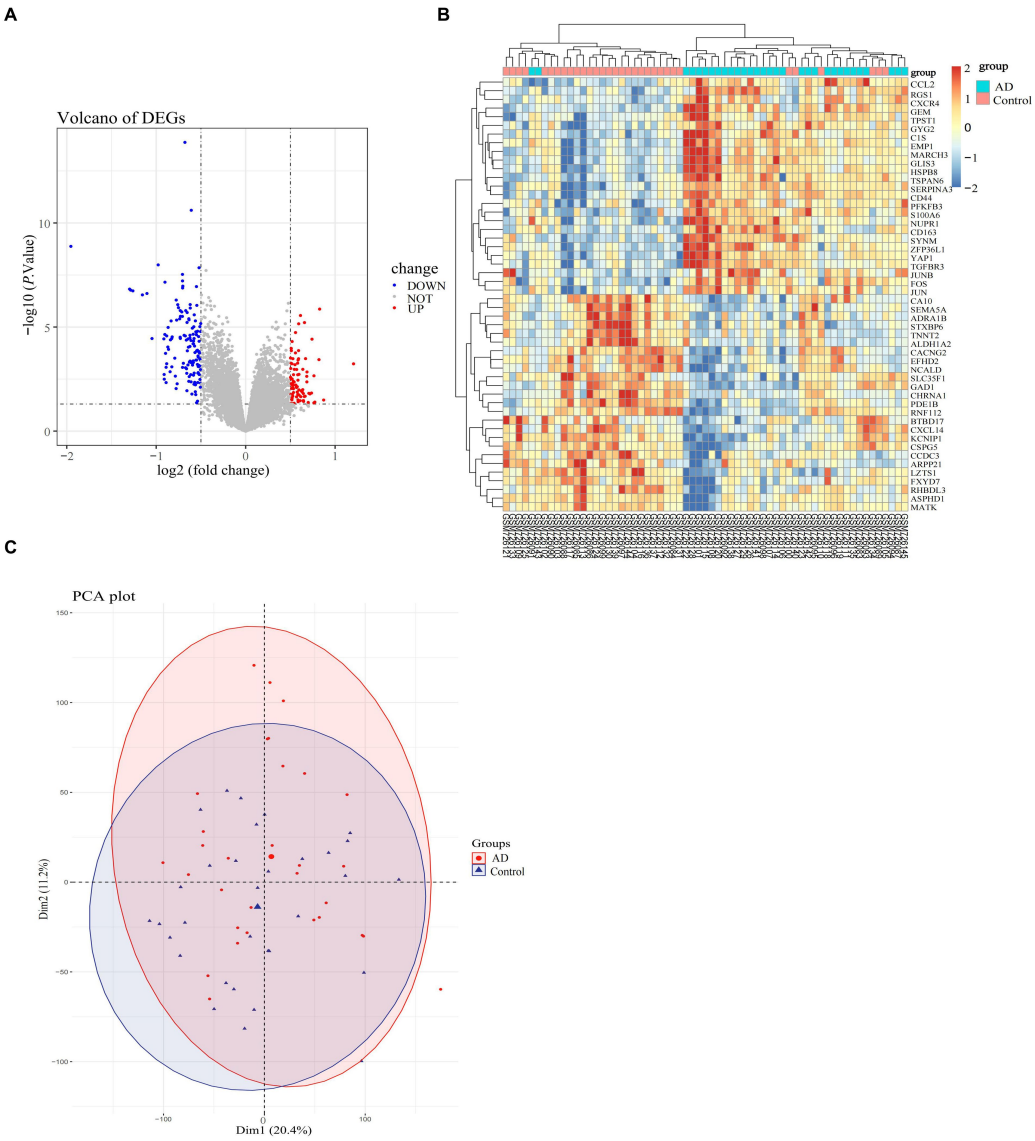
Differential expression profiles of DEGs in the AD-related dataset (GSE29378). **(A)** Volcano plot of selected DEGs, with red color representing up-regulated genes and blue color representing down-regulated genes, and the criteria for selecting DEGs marked with dashed lines. **(B)** Heatmap of selected genes in the samples in the AD and normal groups, with specific color blocks red and blue representing the up-and down-regulation of genes in samples from different groups. The range of variation in color block colors is plotted based on standard scores (Z-scores). **(C)** PCA plots of the overall differences in samples between groups, with confidence ellipses for the distribution of samples in each group indicated by different colors.

The specific screening process for AD-related DEGs is shown in Figure 1. The AD-associated gene expression dataset GSE29378 was retrieved from the GEO database. The total expression of the samples was successfully normalized (Supplementary Figures 1A,B) and then uniformly log_2_FC transformed (Supplementary Figure 1C). The detailed information of the data is shown in Table 1. According to the screening criteria *p* > 0.05 and |log_2_FC| ≥ 0.5, we obtained 225 AD-associated DEGs from GSE29378, of which 85 were up-regulated genes and 140 were down-regulated genes. All DEGs were derived from the comparison of the expression profiles of hippocampal tissues from non-AD patients and AD patients. Both heatmap and volcano plot (Figures 2A,B) showed that the DEGs of hippocampal tissue mRNAs differed significantly between non-AD patients and AD patients. PCA plot (Figure 2C) from data between the non-AD patient group and the AD patient group showed differences in expression patterns between the AD group and non-AD group.

### 3.2 AD-related functional analysis

**Figure 3:**
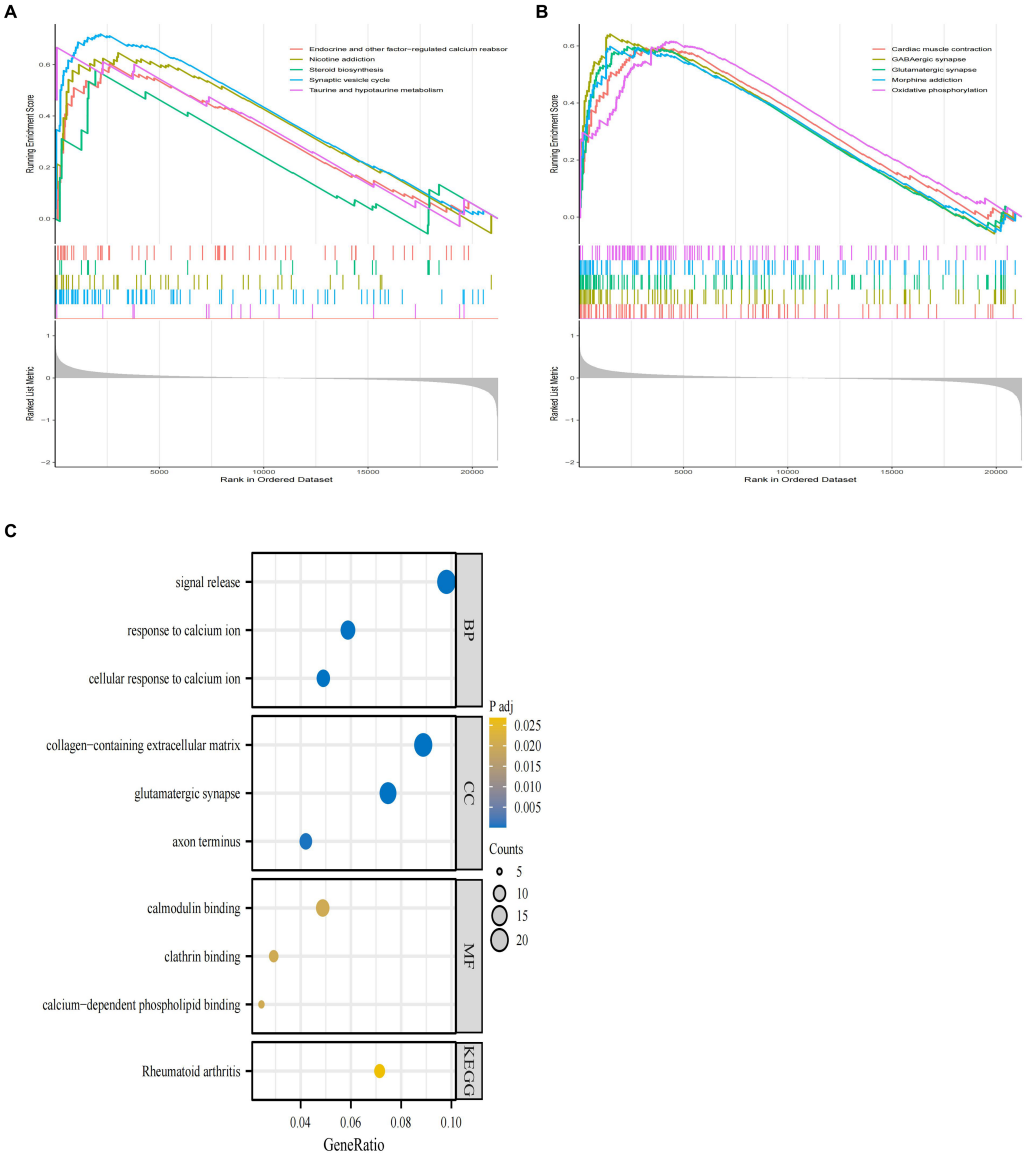
GSEA enrichment analysis of GSE29378 and GO and KEGG analysis of DEGs. **(A,B)** GSEA analysis of the GSEA dataset as a whole with the top ten pathways. **(C)** GO and KEGG pathways of DEGs obtained by screening, with specific pathways categorized as MF, BP, and CC.

In order to further clarify the biological signaling pathways involved in the development of AD, we performed a GESA analysis between the AD and normal groups in GSE29378. This type of analysis was able to identify the overall signaling changes between the groups involved in AD and the normal group. The top ten signaling pathways involved in the AD and normal groups are shown in Figures 3A,B. Among these signaling pathways endocrine and other factors−regulated calcium reabsorb, nicotine addiction, synaptic vesicle cycle, taurine and hypotaurine metabolism, taurine and hypotaurine metabolism, GABA ergic synapse, glutamatergic synapse, morphine addiction and oxidative phosphorylation were clearly upregulated in the AD group.

Meanwhile, we used GO and KEGG analysis to identify the signaling pathways involved in AD-associated DEGs. The results showed that DEGs derived from GSE29378 were mainly enriched in pathways such as cellular response to calcium ion, glutamatergic synapse, and calcium−dependent phospholipid binding (Figure 3C). These signaling pathways may mediate the process of AD development.

### 3.3 Weighting coefficient β value screening and identification of modules in co-expression networks

**Figure 4:**
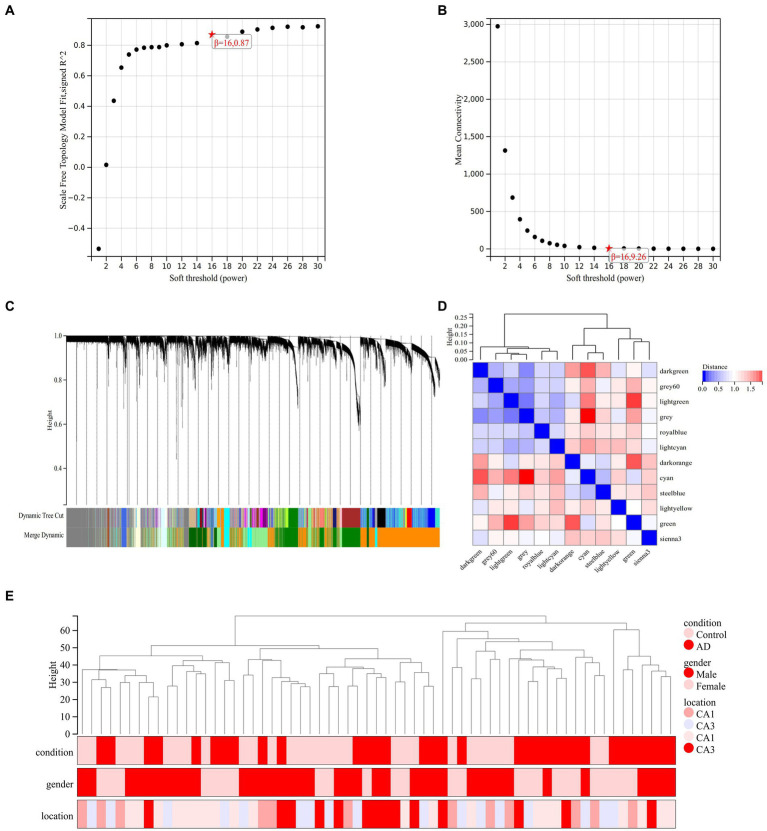
Analysis of co-expression modules. **(A)** Relationship between scale-free fit indices and various soft-threshold powers. **(B)** Relationship between average connectivity and various soft-threshold powers. **(C)** Clustering dendrogram of different genes with different colors representing different modules. **(D)** Vector clustering plot of module features. **(E)** Clustering dendrogram of the 62 samples in GSE29378.

A co-representation network is characterized by a scale-free network is P (k) ∼ k-1, where k denotes the connectivity of the nodes. Therefore, according to the scale-free network rule, the weighting factor β must satisfy the condition that log (k) is negatively correlated with log [P (k)]. In order to determine the value of node connectivity K in a scale-free network, it is necessary to determine the optimal value of the weighting factor β. We transformed the expression profile of GSE29378 into a neighbor-joining matrix to construct TOM, disTOM and gene co-expression networks. The analysis can conclude that the co-expression network is scale-free network when β = 16, R^2^ = 0.87 (Figures 4A,B).

The hierarchical clustering tree was obtained by hierarchical clustering of disTOM (Figure 4C). Then, based on the dynamic tree-cutting method, the minimum number of genes per module was defined as 30, and the intermediate level classification was selected to identify the key clusters. The dissimilarity of module feature genes was calculated under the condition of sensitivity = 3. The tangents of the module tree diagram were selected and the modules with distance less than 0.25 were merged (Figure 4C). Then, the genes that were not classified into any clusters in the previous step were classified into different clusters based on relevance to obtain a total of 12 modules (Figure 4C). One of the gray modules is a set of genes that are considered as a collection of genes that cannot be assigned to any module. The vectors between modules are characterized in Figure 4D. Pearson correlation coefficient was applied to cluster the samples to obtain the sample clustering tree shown in Figure 4E.

### 3.4 Screening and extraction of co-expression modules of AD trait-related genes

**Figure 5:**
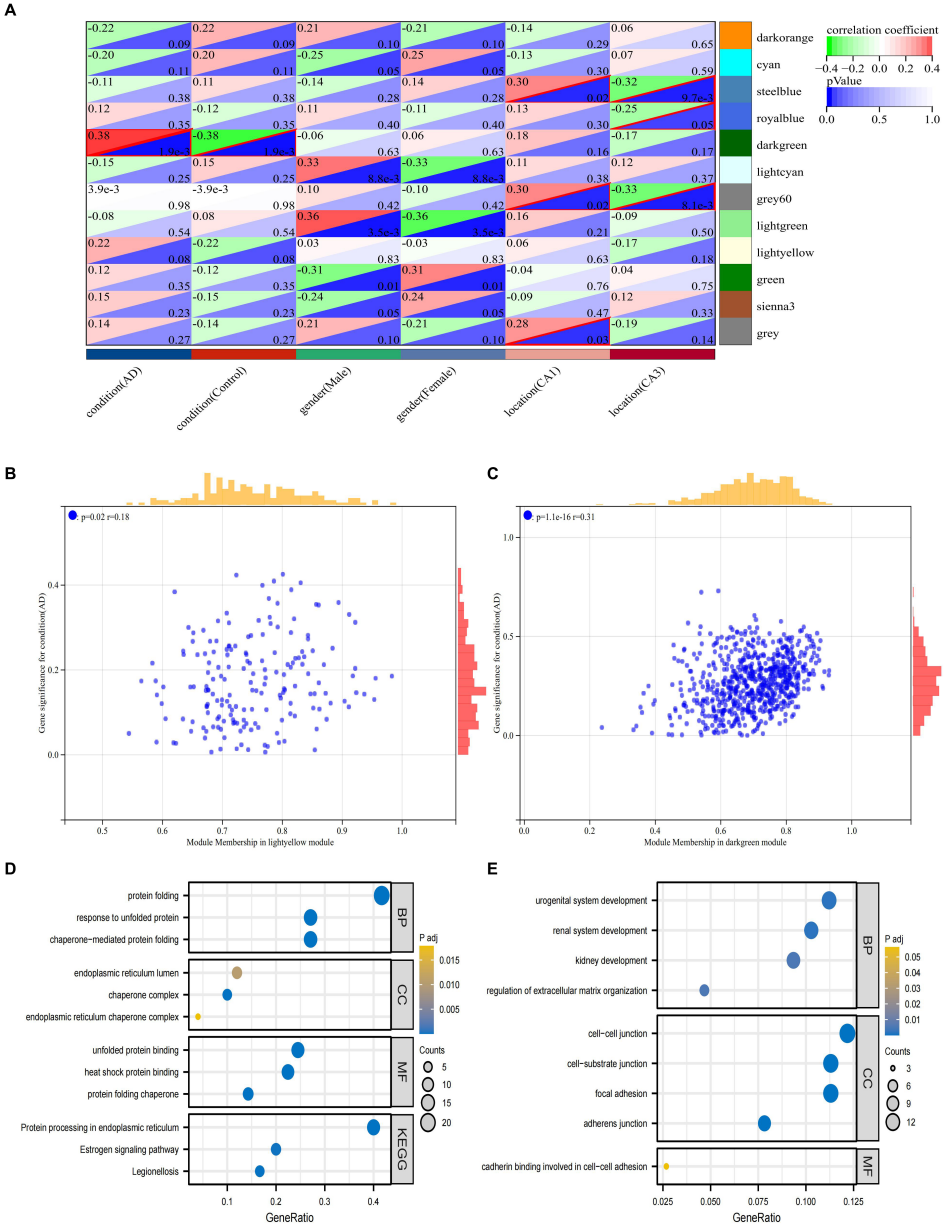
Analysis of clinically important modules. **(A)** Relationships of the 6 features with the 12 modules; **(B)** Scatterplot describing the relationship between MM and GS in the dark green module; **(C)**. Scatterplot describing the relationship between MM and GS in the bright yellow module. **(D)** GO and KEGG analysis of genes in the dark green module. **(E)** GO and KEGG analysis of genes in the dark green module.

The first principal component in each module is termed ME, which is the single value that represents the highest percentage of variation among all gene expression values. Pearson correlation coefficients and clinical information were calculated for the MEs of all modules to determine which modules were associated with clinical features such as the development of AD and its distribution in hippocampal CA1 and CA3 brain regions (Figure 5A). The dark green module was significantly associated with the AD group (R = 0.38, *p* = 1.9e-3) and the control group (R = −0.38, *p* = 1.9e-3). The gray 60 module and the steel blue module were significantly associated with the CA1 brain region (gray 60: R = 0.3, *p* = 0.02, steel blue: R = 0.3, *p* = 0.02) and the CA3 brain region (gray 60: R = −0.33, *p* = 8.1e-3, steel blue: R = −0.32, *p* = 9.7e-3). The royal blue module was significantly associated with the CA3 brain region (R = −0.25, *p* = 0.05). Each module represents a specific clinical feature of AD patients and the highly co-expressed genes in the same module have potential biological significance.

To ensure the reliability of the identification results of the AD-associated modules, these modules were re-identified by calculating the average absolute gene significance (GS) values of the AD-associated genes in each module. The dark green module and the bright yellow module had the largest absolute mean values of GS associated with AD (Figures 5B,C). Hub gene screening requires the identification of module membership (MM) to determine the correlation between gene and given module. Therefore, the MM value for each gene needs to be calculated to identify the genes in the module. Using MM threshold = 0.8, GS threshold = 0.1, and weight threshold = 0.1 criteria, we obtained 124 and 52 genes in dark green and bright yellow modules, respectively. These genes had the highest correlation with AD and may have an important relationship with the development of AD. To clarify the functions of these two groups of genes, GO and KEGG analyses were performed, respectively. The genes in dark green module were mainly associated with protein folding, response to unfolded proteins, endoplasmic reticulum chaperone complexes, and protein processing in the endoplasmic reticulum and other pathways (Figure 5D). The genes in the bright yellow module were mainly enriched in pathways such as regulation of extracellular matrix organization, intercellular junctions, genesis of the reproductive system, and calcineurin binding involved in intercellular adhesion (Figure 5E).

### 3.5 Identification of hub genes associated with AD

**Figure 6:**
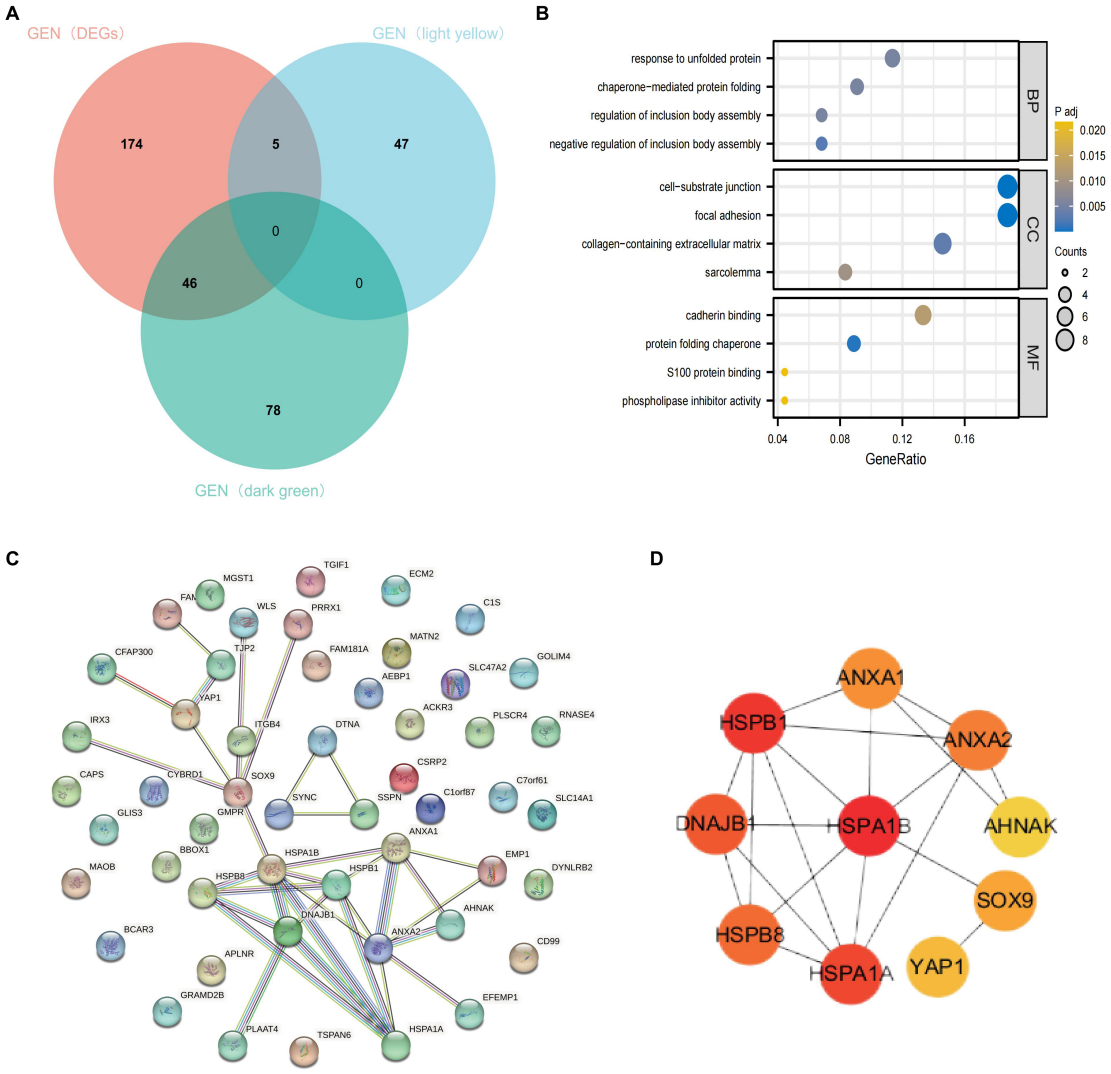
Identification of hub genes. **(A)** Intersection of DEGs, bright yellow module genes and dark green module genes. **(B)** Functional enrichment analysis of the intersected genes. **(C)** PPI network of intersecting genes. **(D)** Calculated top ten ranked hub genes.

To further clarify the important hub genes mediating the development of AD, we obtained AD-associated DEGs intersected with dark green module genes and bright yellow module genes, respectively, and a total of 51 genes were obtained (Figure 6A). GO and KEGG analyses indicated that these intersected genes were mainly enriched in the response to unfolded proteins, cell-lining junction, calcium adhesion protein binding, and chaperonin-mediated protein folding pathways (Figure 6B). These 51 genes were put into the string database to obtain the interaction networks between their corresponding proteins (Figure 6C). Ten most closely linked genes (HSPA1B, HSPB1, HSPA1A, DNAJB1, HSPB8, ANXA2, ANXA1, SOX9, YAP1 and AHNAK) in the network data were extracted and identified as the hub genes of AD using the huba algorithm of cytoscape software (Figure 6D). They have important roles in the development of AD.

### 3.6 Revalidation of AD-related hub genes

**Figure 7:**
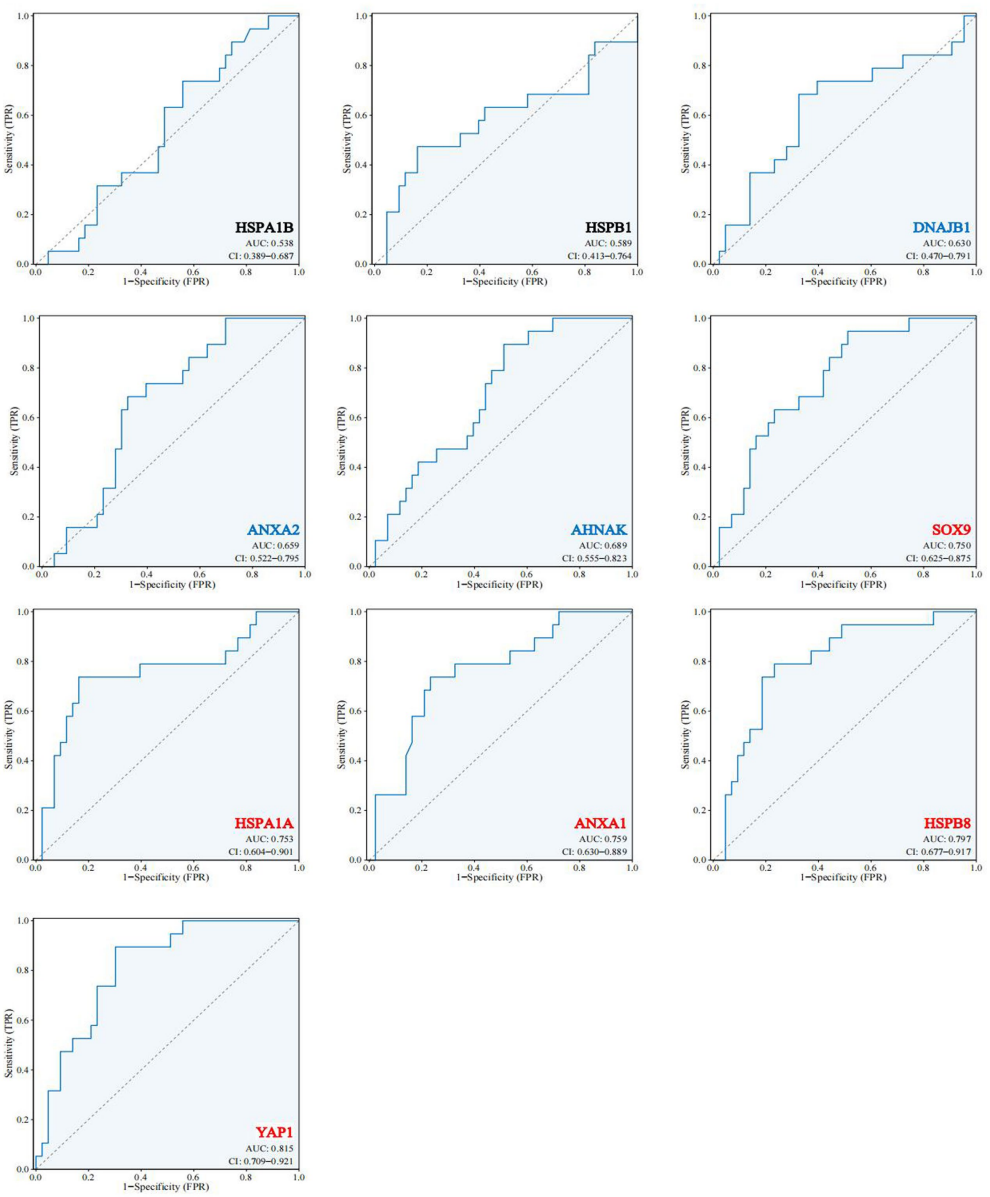
Expression and functional validation of hub genes. 10 hub genes in the validation dataset GSE48350 ROC profile.

To test whether the 10 hub genes have a diagnostic role in the development of AD. We extracted another AD-related dataset (GES48350) as a validation cohort. The expression of these 10 hub genes was used as a diagnostic indicator to predict the occurrence of AD or not, and the corresponding ROC curves were made. As shown in Figure 7 among these 10 hub genes, HSPA1B and HSPB1 have weak diagnostic efficacy for AD (AUC < 0.6), DNAJB1, ANXA2 and AHNAK have some diagnostic efficacy for AD (0.6 < AUC < 0.75), while SOX9, HSPA1A, ANXA1, HSPB8 and YAP1 have significant diagnostic efficacy for AD (AUC > 0.75). The results suggest that external validation of these 10 hub genes indeed predicts the development of AD and may have an important role in the development of AD.

## 4 Discussion

AD is the most common cause of dementia, with 60–80% caused by genetic factors (Scheltens et al., 2021). Since the core mechanisms of molecular characterization and mechanistic pathways are still unknown, more precise pathogenic targets and key pathways can be obtained by using multiple integrated analytical methods to covary the histological data of AD. This may not be achievable with any single histological research method. Therefore, we aimed to jointly identify novel AD hub genes by analyzing DEGs and WGCNA for transcriptomic data information in the hippocampal region of AD.

Transcriptomic information from lesions in many brain regions should be taken into account in the screening of DEGs for AD, including: posterior cortical atrophy for visuospatial impairments (Alladi et al., 2007), frontotemporal lobe degeneration for progressive aphasia and cognitive deficits (Bergeron et al., 2018; Graff-Radford et al., 2021), frontal neuroprogenitor fiber tangles for executive deficits (Townley et al., 2020), corticobasal degeneration for motor dysfunction (Mathew et al., 2012), and hippocampus for learning and memory deficits (Mu and Gage, 2011). AD is often characterized by progressive memory loss and cognitive deterioration mostly related to hippocampal function, and the hippocampus as a specific, vulnerable brain region is often the first to be affected by AD lesions. Therefore, we chose to compare the transcriptomic information (GSE29378) of normal human hippocampal brain regions and hippocampal brain regions of AD patients to uncover AD-related hub genes.

In the GSE29378 dataset, we compared AD and non-AD expression data with the help of differential genes to find AD-related DGEs (*p* < 0.05, |log2FC| ≥ 1). In the exploration of DEGs associated with previous diseases, the screening criteria for DEGs in brain tumors such as gliomas were mostly set at *p* < 0.05, |log2FC| ≥ 1.0 (Huang et al., 2020), and the screening criteria for neurological autoimmune diseases such as multiple sclerosis were also mostly set at this criterion (Li et al., 2021). However, after downloading and standardizing the data, it was difficult to obtain a sufficient number of DEGs associated with AD even at *p* < 0.05. When screening for differential genes, we initially chose to use *p* < 0.05, |log*_2_* FC| ≥ 1, but only got 8 differential genes. This suggests that we may have missed some important genes, and at the same time we cannot guarantee the novelty of those genes found. This is likely because mRNAs in the hippocampus are not as up-regulated or down-regulated in AD as in tumors and autoimmune diseases, or AD is a chronic process with complex gene involvement and ack of large gene expression in quantity. It is difficult for the genes to be involved in the same time to directly regulate the AD process and lack specificity. The screening criteria for the AD dataset have not yet been determined. A and B use |log2FC| ≥ 0.5, *p* < 0.05 as the criteria for screening DEGs (Wang et al., 2021; Zhang et al., 2021). In order to ensure that a sufficient number of DEGs were obtained, we also chose this criterion for screening DEGs. However, this kind of screening will inevitably miss some important genes affecting the occurrence and development of AD. Biological pathways involved in altered synaptic vesicle cycling, GABAergic synapses, and glutamatergic synapses are associated with altered traits between groups of AD and non-AD samples in GSE29378. Previous studies have shown that the function of neuronal axonal transport and synaptic vesicle release in neurons determines neuronal plasticity (Lazarevic et al., 2017; Palikaras and Tavernarakis, 2020). In contrast, the regulation of Aβ through CDK5, calmodulin phosphatase signaling, and increased synaptic vesicle cycling in the AD state disrupts synaptic function and homeostasis, ultimately leading to cognitive decline and neurodegeneration (Wu et al., 2022). At the same time, APP, the precursor protein of Aβ, interacts with the neuron-specific potassium chloride (K + -Cl-) cotransporter KCC2/SLC12A5 and regulates GABAergic synaptic levels and activity affecting synaptic vesicle release (Tang, 2019). Compensatory enhancement of excitatory signaling or remodeling in early AD correlates with an early and pronounced loss of glutamatergic synapses in its progression (Mitew et al., 2013). This suggests that the differences we defined between the AD and normal groups are indeed associated with AD, and after screening for DEGs, these DEGs were mainly enriched in the pathways of calcium-dependent phospholipid binding, glutamatergic synapse, and cellular response to calcium ion in line with the GESA analysis, which also suggests that these DEGs are indeed associated with AD.

However, a single approach still struggles to explain the importance of these 57 DEGs for AD, and we used the WGCNA analysis to avoid using *p*-values and logFC alone as the sole measure of hub genes. WGCNA is not only a systems biology approach for describing patterns of genetic correlation among microarray samples, using module signature genes to summarize these sample clusters, but also a signature gene network approach to correlate modules with each other and with external sample traits, as well as for calculating module membership metrics (Langfelder and Horvath, 2008; Zhao et al., 2010). Thus, this methodology is more of a holistic methodology that measures the co-expressed gene modules among samples and explores the linkages between these modules and sample traits. The performance of WGCNA in hub discovery for AD and other neurological disorders was well supported by the literature (Mukherjee, 2021; Lin et al., 2022). However, we must also realize that there are limitations in WGCNA analysis: 1. The samples in the selected dataset should be larger than 15 in order to focus on the formation of effective modules. 2. WGCNA mostly uses default parameters to control the construction of the network and the extraction of the different modules, and does not preset the differentially expressed genes in advance. After our WGCNA analysis of the AD dataset, we focused on a few specific modules: the dark green module and the bright yellow module, which are positively correlated with AD status, the gray 60 module and the steel blue module, which are positively correlated with CA1, as well as the navy blue, steel blue, and gray 60 modules, which are negatively correlated with the CA3 brain region. However, we considered the hub genes should be more used for AD diagnosis and treatment, the relationship between the module genes and hippocampal subdivisions and their role on AD will be further elucidated in subsequent studies. One of the dark green modules we extracted 124 genes, these are mainly related to the response to unfolded proteins. The genes in bright yellow module, on the other hand, were mostly involved in intercellular adhesion, organ development, and other energies. This showed the consistency of processing with the development of AD occurrence. Finally, we obtained a total of 52 genes by intersecting these module genes with the DEGs of AD, and further analysis showed that these genes were associated with protein folding and intercellular adhesion, corroborating the previous analysis. Finally, we obtained 10 hub genes among them: HSPA1B, HSPB1, HSPB8, HSPA1A, DNAJB1, ANXA2, ANXA1, SOX9, YAP1 and AHNAK.

10 AD-related hub genes were eventually identified, and these were supported in the literatures that some of them are involved in the development of AD. Five of these 10 hub genes (HSPA1B, HSPA1A, HSPB1, HSPB8, DNAJB1) are from the heat shock protein family. Heat shock proteins (HSPs) are molecular chaperones which can be categorized into nine subfamilies (HSP10 (HSPE), HSP20 (HSPB), HSP40 (DNAJA, DNAJB, DNAJC), HSP60 (HSPD), HSP70 (HSPA), HSP90 (HSPC), and the large HSPs) with forms and functions capable of being expressed and induced by stress to promote proper folding of newly synthesized polypeptides, regulate assembly and disassembly of multiprotein complexes, and regulate intracellular protein trafficking and transmembrane transfer (Tower, 2009; Hu et al., 2022). It was found that the A2 allele of HSPA1B was able to quantitatively affect the mental state of patients and attenuate the anti-cellular oxidative stress effect of HSPA1B, increasing the incidence of AD (Clarimón et al., 2016; Ramos et al., 2018). HSPA1A is an isoform of HSP70. HSPA1A was found to be highly expressed in cerebrospinal fluid extracellular vesicles of AD patients directly involved in AD progression. HSPA1A expression was down-regulated in the prefrontal cortex of patients with advanced AD, and these may be directly related to its inhibition of the aggregation of tau protein isoforms (Voss et al., 2012; Kitzlerová et al., 2018; Muraoka et al., 2020). Moreover, it has also been shown that HSPA1A is also a gene that mediates the interaction between AD and depression (Mathys et al., 2019). HSPB8 also has an important effect on the development of AD. In cell lines expressing amyloid precursor protein (AβPP), HSPB1 expression alters the expression and processing of AβPP and directly reduces the amount of Aβ42 released by the cell line protecting the cells from the potential toxic effects of Aβ (Conway et al., 2014). At the same time HSPB1 can segregate toxic Aβ oligomers and convert them into large non-toxic aggregates to eliminate the toxicity of Aβ oligomers to cells, potentially reducing plaque deposition in AD patients (Ojha et al., 2023). Inhibition of the lncRNA SNHG14/UPF1 axis promotes HSPB8 expression, which inhibits apoptosis in AD neuronal cells (Tan et al., 2023). Meanwhile, HSPB8 can inhibit the production of D-Aβ1-40 and the formation of β-folds, and completely inhibit D-Aβ1-40-mediated cerebrovascular cell death (Wilhelmus et al., 2006). The DNAJB (Hsp40) family functions in protein folding or defolding, membrane trafficking, synaptic regulation, and mitochondrial function affect not only dopaminergic neurotransmission, but also Parkinson’s-associated neuropathological changes (Hasegawa et al., 2018). At the same time this chaperone protein interacts with β-proteins to promote intracellular aggregation of β-peptides and facilitates their translocation to mitochondria to exert toxic effects on AD patients (Ring et al., 2022). Annexin A1 (ANXA1) is a glucocorticoid anti-inflammatory mediator in the peripheral system that efficiently and selectively removes apoptotic neuron-like cells (McArthur et al., 2010). In early AD, ANXA1 is increased in the brain, where it is able to reduce Aβ levels by increasing the enzymatic degradation of neprilysin in N2a cells and to stimulate phagocytosis of Aβ by microglia to reduce inflammatory mediators produced as a result of Aβ stimulation (McArthur et al., 2010). ANXA1 levels were reduced in the peripheral plasma of patients with behavioral variants of AD and showed a correlation between ANXA1 and the production and abatement of its peripheral inflammatory mediators (Fraga et al., 2019). We found that ANXA2 did not exhibit a direct association with AD, but it has been shown that ANXA2 develops calcium-regulated membrane-cytoskeletal junctions that exhibit interactions with tau proteins in the context of Ca*^2+^* segregation or elimination of differential capture of tau proteins by knockdown of ANXA2 (Gauthier-Kemper et al., 2011). SRY-box transcription factor 9 (SOX9) is a positive regulator of astrocyte formation, and activation of miR-22-3p levels in the hippocampus of mice improves their cognitive performance through SOX9-mediated activation of the NF-κB signaling pathway (Xia et al., 2022). Yes-associated protein 1 (YAP1), a transcriptional regulator that promotes tissue growth and regeneration, is also a potential regulator of AD (Xu et al., 2017). YAP was down-regulated and inactivated in hippocampal astrocytes of AD model mice in a hippocampal pathway-dependent manner, whereas activation of the YAP-CDK6 pathway improved cognitive function in both AD model mice and senescent mice (Ries et al., 2016). In the study of cognitive improvement by dexmedetomidine (Dex) in AD patients, the miR-129/YAP1/JAG1 axis may be a potential mechanism by which Dex protects against cognitive impairment in AD patients (Sun et al., 2020). However, we found that only 9 of these 10 hub genes were found to be directly associated with AD, among which the role of HNAK in AD has not yet been elucidated. Meanwhile, existing studies on the mechanisms of these molecules for the development of AD are still small and vary in depth, and these molecules still have great potential for research.

**Figure 8:**
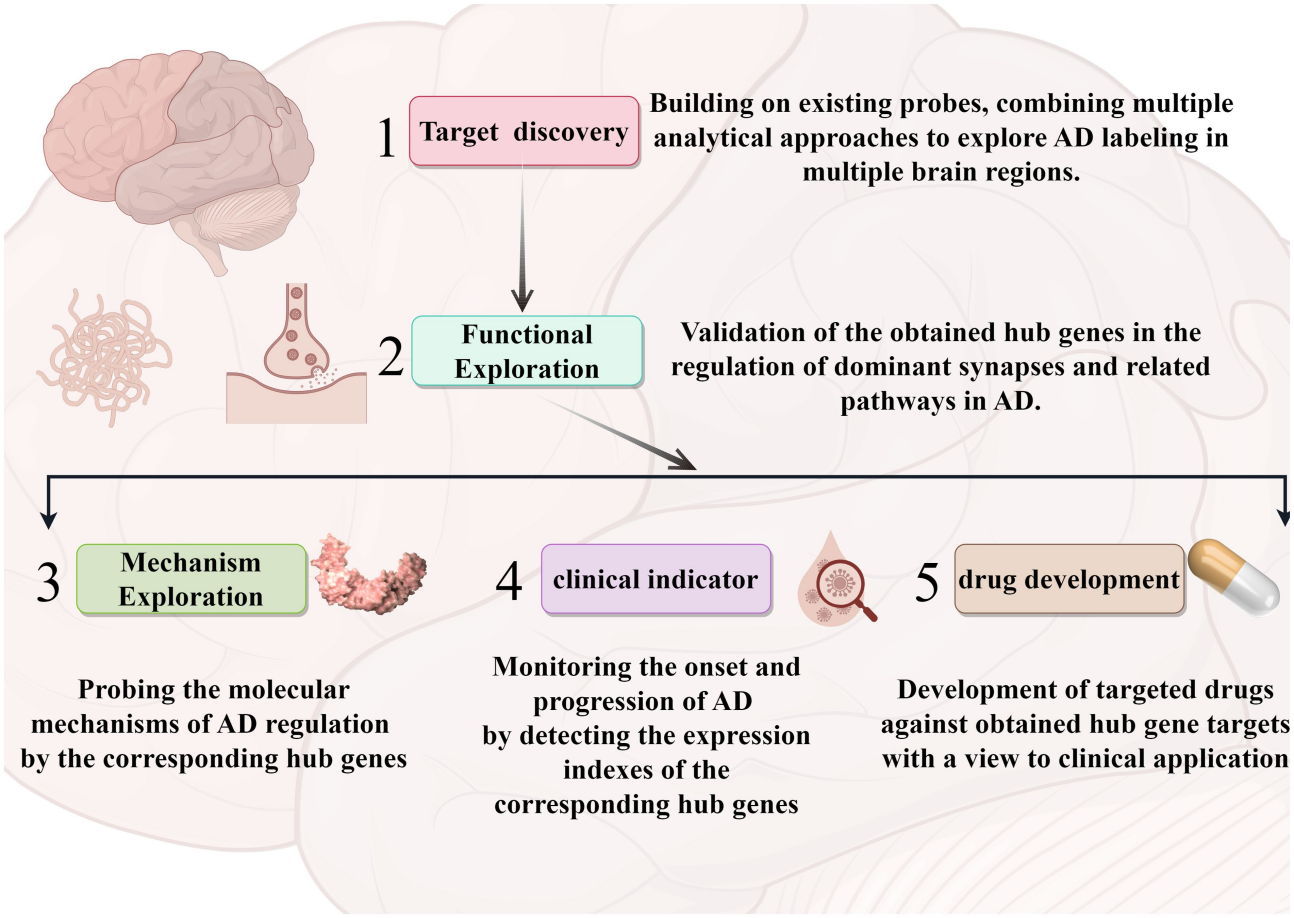
A roadmap for follow-up research. The arrows represent the corresponding process sequence. Different modules represent different analyses and applications. The graphics were created with the help of the Figdraw tool.

In the past literature, there is the identification of AD-related hub genes by using only the modular genes in DEGs or WGCNA to identify AD hub genes, which is still slightly insufficient in terms of in data selection and processing (Wu et al., 2021; Liang et al., 2022). In comparison, the strengths in our study: 1. The combination of two common methods can minimize the selection bias on hub genes. 2. Step-by-step analysis and validation, and try to be biologically functional and clinically phenotypically oriented in screening. 3. Supplementary dataset as a validation cohort for secondary validation of clinical value. However, this study still has some limitations that are difficult to avoid. Firstly, due to the difficulty in obtaining tissue samples, the active state of the tissue cells cannot be guaranteed in subsequent studies. Secondly, the present study was limited to the hippocampal region only, whereas the pathogenesis of AD is related to multiple brain regions, and a single role of the hippocampus cannot explain the multifactorial problem of AD. Meanwhile, since our criteria for screening hub genes were decided based on the existing analysis, there may still be some neglected important factors involved in the development of AD. In our subsequent studies we will combine different brain regions for further and more favorable analysis and validation. By targeting some new key targets and pathogenic pathway pathways (vascular accidents and specific functional synapses), effective treatment of AD may be realized (Shekhar et al., 2021). Specifically, the follow-up studies, as shown in Figure 8, consisted of combining a multi-omics approach to probe AD-related hub genes within multiple brain regions based on the present study. Analyze and validate the roles of these hub genes in the dominant pathways and synapses. Based on this, experimental models were established to investigate the molecular mechanisms of AD regulation by these molecules. We will also explore the role of these molecules in predicting the course of the disease in the clinic and develop possible target drugs.

In conclusion, we used a combination of differential gene screening methods and WGCNA sorting of modular genes to jointly identify hub genes that mediate AD onset and development in hippocampal brain regions. Ten hub genes, HSPA1B, HSPB1, HSPA1A, DNAJB1, HSPB8, ANXA2, ANXA1, SOX9, YAP1, and AHNAK, were identified by extracting genes intersecting the two modular genes in the DEGs and WGCNA. an external dataset validated the diagnostic significance of these molecules for AD. This study identifies new AD-related genes in the hippocampus and provides new potential therapeutic biomarkers, and molecular pathways.

## Data availability statement

The datasets presented in this study can be found in online repositories. The names of the repository/repositories and accession number(s) can be found in the article/Supplementary material.

## Author contributions

YC: Conceptualization, Data curation, Methodology, Software, Writing – original draft, Writing – review & editing. ZL: Investigation, Methodology, Writing – review & editing, Writing – original draft. XG: Formal analysis, Project administration, Writing – review & editing. HL: Data curation, Validation, Writing – review & editing. ZG: Funding acquisition, Project administration, Resources, Writing – review & editing.
