# Supplementary material for: Identification of novel hub genes for Alzheimer’s disease associated with the hippocampus using WGCNA and differential gene analysis
Source: Front Neurosci. 2024 Mar 7;18:1359631. doi: 10.3389/fnins.2024.1359631 (PMC10954837; doi:10.3389/fnins.2024.1359631)
Supplement: Supplementary file 1 [file Data_Sheet_1.PDF]

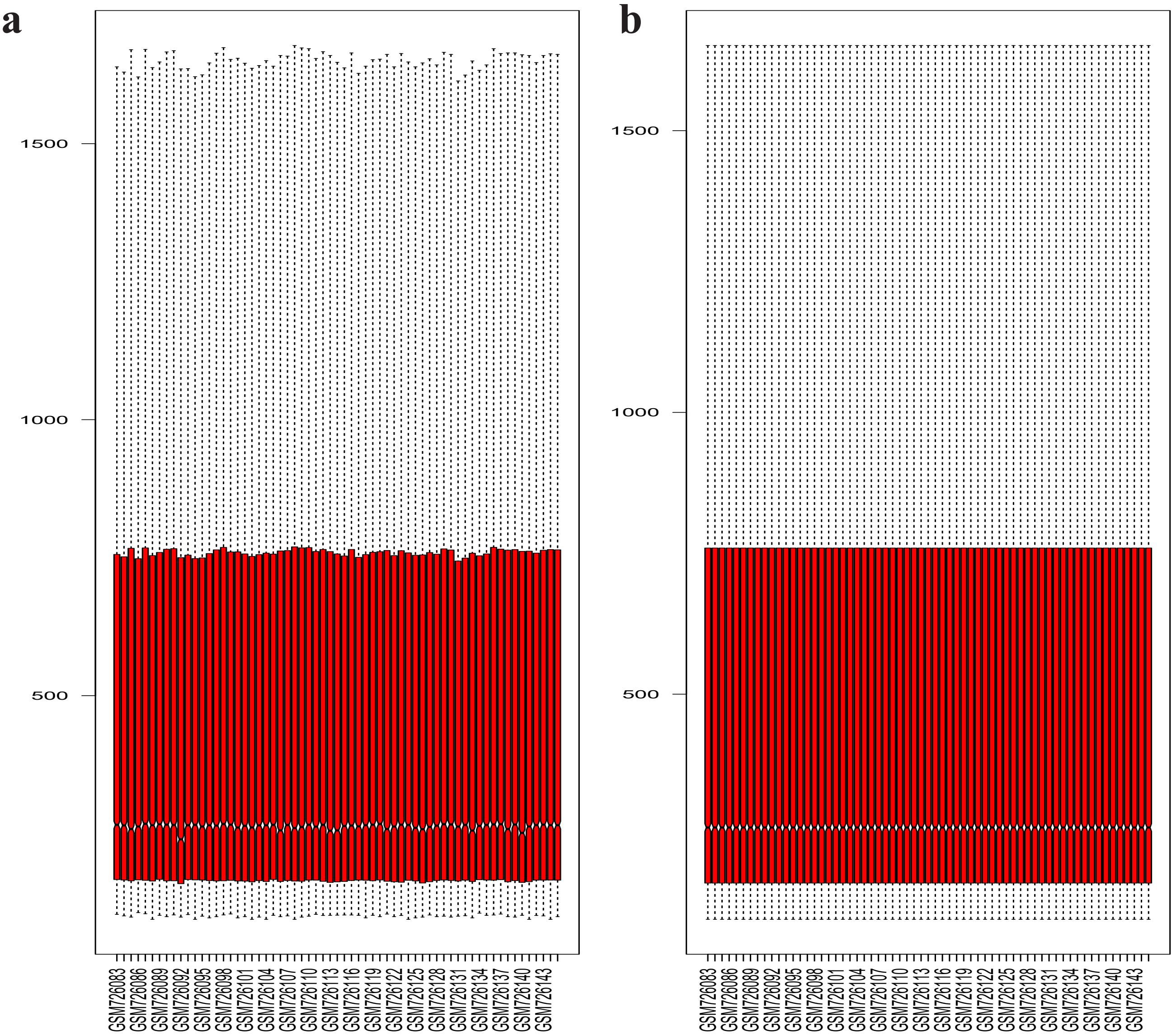

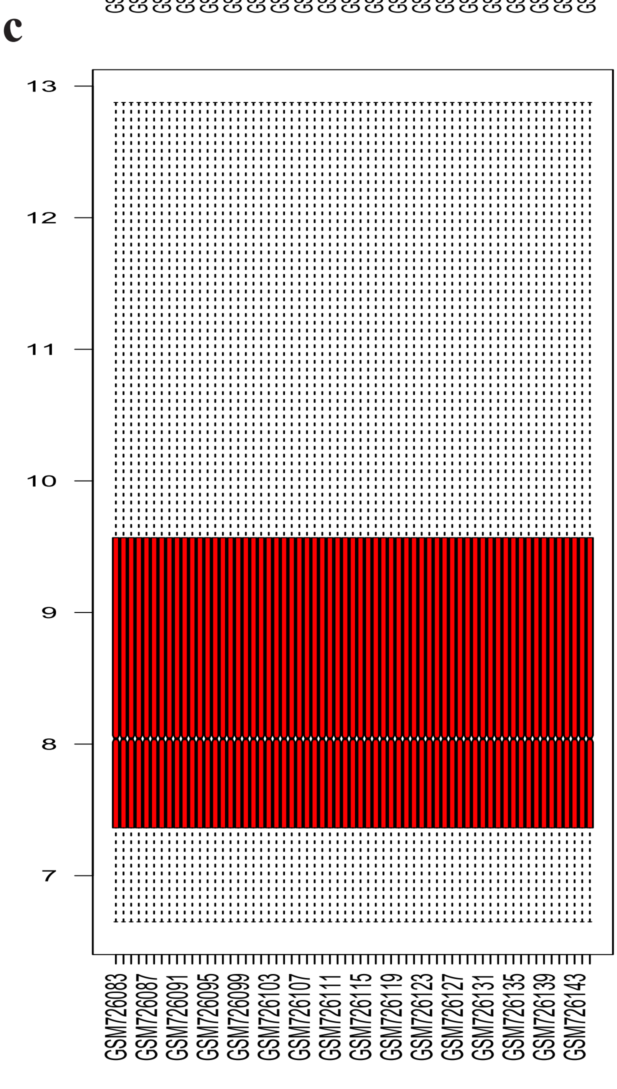

Supplement figure 1.Standardization of data. (a) Total mRNA expression for each sample in the GSE29378 dataset that was not processed. (b) Total mRNA expression of each sample in the GSE29378 dataset after correction. (c) Expression of each sample after log_2FC transformation.
