# Supplementary material for: Identification of novel hub genes for Alzheimer’s disease associated with the hippocampus using WGCNA and differential gene analysis
Source: Front Neurosci. 2024 Mar 7;18:1359631. doi: 10.3389/fnins.2024.1359631 (PMC10954837; doi:10.3389/fnins.2024.1359631)
Supplement: Supplementary file 2 [file Data_Sheet_2.PDF]

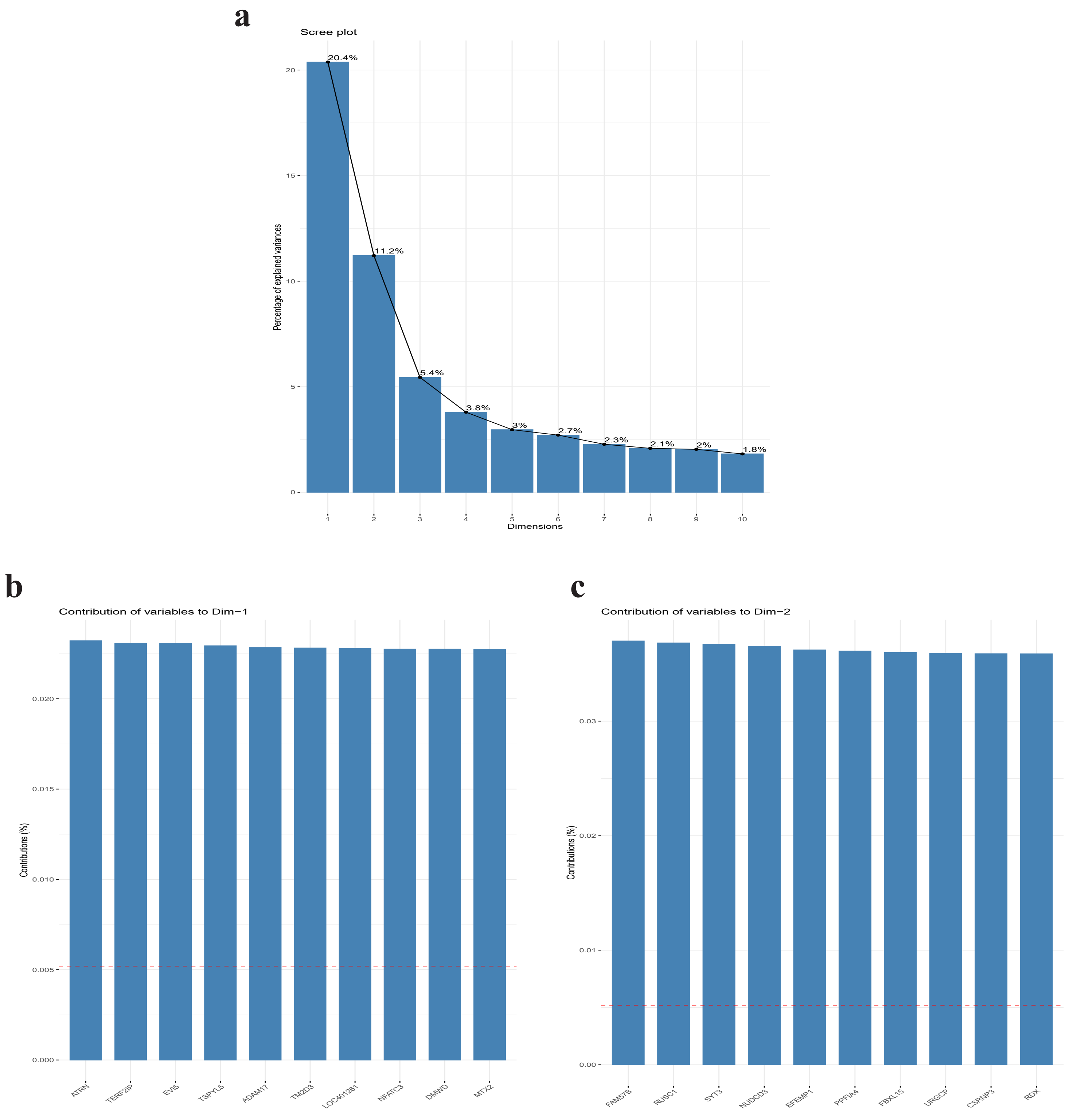

Supplementary figure 2. Eigenvalue profile of PCA. (a) Top 10 eigenvalues. (b) Amount of contribution of the top ten ranked genes to PC1. (c) The amount of contribution of the top ten ranked genes to PC2.
