# Supplementary material for: Identification of novel hub genes for Alzheimer’s disease associated with the hippocampus using WGCNA and differential gene analysis
Source: Front Neurosci. 2024 Mar 7;18:1359631. doi: 10.3389/fnins.2024.1359631 (PMC10954837; doi:10.3389/fnins.2024.1359631)
Supplement: Supplementary file 3 [file Data_Sheet_3.PDF]

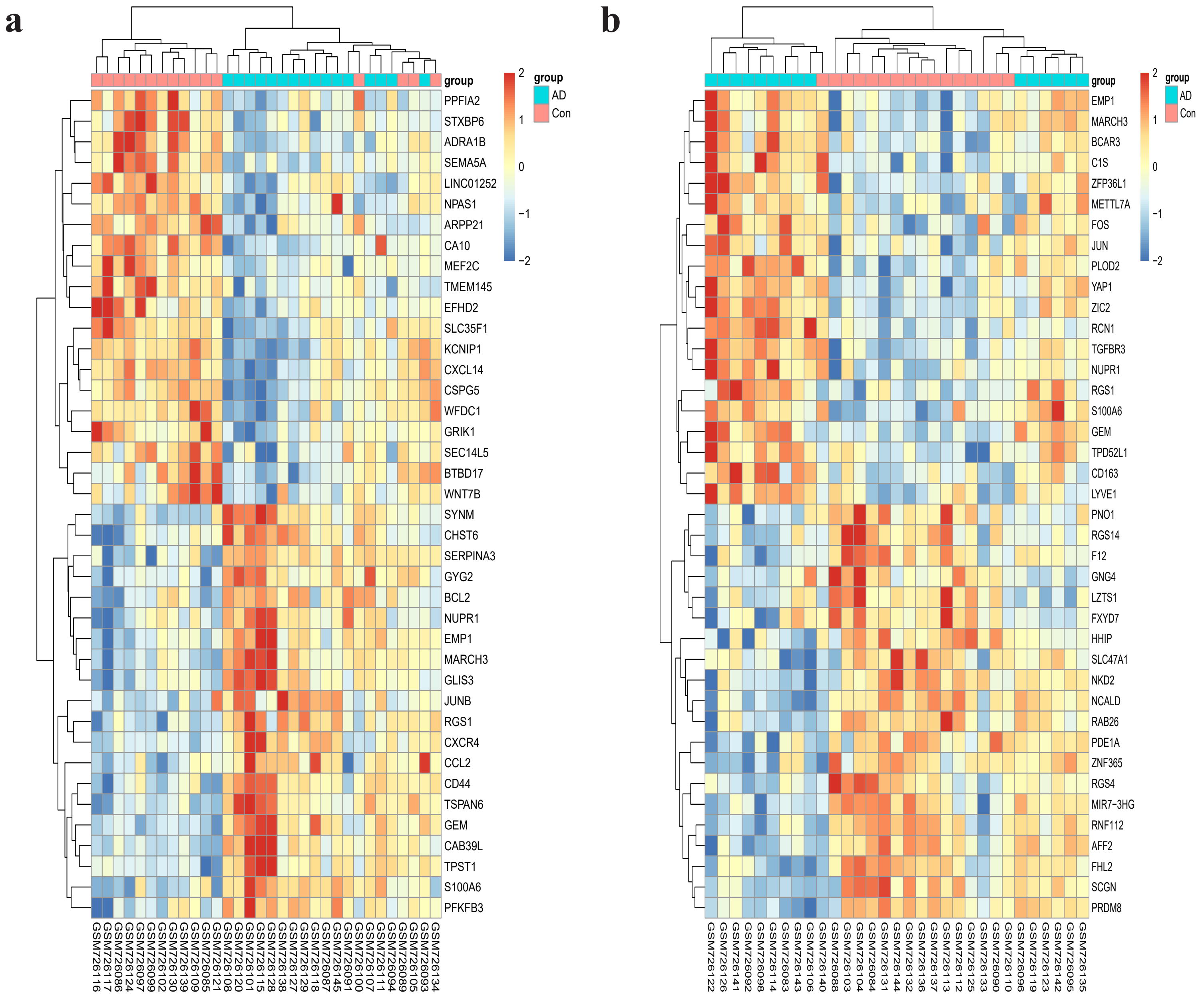

Supplementary figure 3. Heatmap of genes in GSE29378 in CA1 and CA2 brain regions (a) Expression of selected genes in CA1 brain region in AD and normal groups. (b) Expression of selected genes in CA2 brain regions in AD and normal groups.
